# Preparation of a Highly Sensitive Electrochemical Aptasensor for Measuring Epirubicin Based on a Gold Electrode Boosted with Carbon Nano-Onions and MB

**DOI:** 10.3390/bios12121139

**Published:** 2022-12-07

**Authors:** Esmail Sohouli, Masoumeh Ghalkhani, Tahereh Zargar, Farhad Ahmadi

**Affiliations:** 1 Electrochemical Sensors Research Laboratory, Department of Chemistry, Faculty of Science, Shahid Rajaee Teacher Training University, Lavizan, P.O. Box 16785-163, Tehran 167881-5811, Iran; 2 Physiology Research Center, Iran University of Medical Sciences, P.O. Box 14665-354, Tehran 141663-4793, Iran

**Keywords:** epirubicin, carbon nano-onion, electrochemical aptasensor, gold electrode

## Abstract

Epirubicin is prescribed as an essential drug for treating breast, prostate, uterine, and gastrointestinal cancers. It has many side effects, such as heart failure, mouth inflammation, abdominal pain, fever, and shortness of breath. Its measurement is necessary by straightforward and cheap methods. The application of aptamer-based electrochemical sensors is accounted as a selective option for measuring different compounds. In this work, a thiol-modified aptamer was self-assembled on the surface of the gold electrode (AuE) boosted with carbon nano-onions (CNOs), and coupled with methylene blue (MB) as an electroactive tracker to achieve a sensitive and selective aptasensor. In the absence of the epirubicin, CNOs binds to the aptamer through a π-π interaction enhancing the MB electrochemical signal. When epirubicin binds to the aptamer, the adsorption of CNOs and MB to the aptamer is not well established, so the electrochemical signal is reduced, consequently, the epirubicin value can be measured. The prepared aptasensor demonstrated an excellent sensitivity with a curve slope of 0.36 μI/nM, and 3 nM limit of detection in the linear concentration range of 1–75 nM. The prepared aptasensor was accurately capable of measuring epirubicin in blood serum samples.

## 1. Introduction

Epirubicin (EP) is an anticancer drug that interferes with the growth and spread of cancer cells in the body. Epirubicin and its medicinal derivatives are used to treat breast and prostate cancer. The action mechanism of this drug is based on its inhibiting effect on the synthesis of nucleic acids (DNA and RNA) and proteins [1,2,3]. This drug is one of the cytotoxic agents of anthracyclines. Anthracyclines have also been found to interfere with some biochemical and biological functions in eukaryotic cells. After intravenous administration, epirubicin is rapidly and widely distributed in tissues, and about 77% binds to plasma proteins, mainly albumin [4,5]. Epirubicin is metabolized quickly in the liver and other organs and cells, including red blood cells. This drug and its primary metabolite are excreted mainly through bile and a tiny amount through urine. The epirubicin uses causes some side effects and its overdose leads to cardiotoxicity. Fatal congestive heart failure may also occur during treatment by epirubicin, even months or years after the end of drug consumption. Also, in some cases, it leads to a severe weakening of the bone marrow in the patient [6,7]. Since the long-term use and high dose of the drug can be very dangerous for the patient, determining the concentration of epirubicin in human biological fluids is helpful in optimizing drug doses in the treatment of cancer cells. So far, important analytical methods and techniques have been used to determine the concentration of this drug in biological samples [8,9,10,11,12]. Recently, electrochemical methods have been widely used to measure epirubicin due to their simplicity, high sensitivity, and low cost [13,14,15,16,17,18,19].

One of the most important carbon nanomaterials that are recently used in electrochemical measurements and surface modification methods is the carbon nano-anions (CNOs). CNOs are actually formed of multi-layered fluorine structures, which their shape, size, and number of layers depend on the applied synthesis method. The utilization of the CNOs as a suitable material for solid electrodes modification has been reported for the glassy carbon and gold electrodes [20,21,22,23]. Previous studies confirmed boosting the electron exchange at the surface of the electrodes modified with carbon nanomaterials, especially CNOs, originated from their high surface area, very good electrical conductivity, high adsorption capacity, and accessible active sites [24,25]. Also, the low toxicity of these compounds compared to other nano-based compounds causes the use of these nanomaterials to be considered in the fields of pharmaceutical diagnosis and biomedicine. Since aptamers are single strands of DNA or RNA molecules that bind specifically to the target molecule [26,27], the use of these types of molecular detection elements improves the sensitivity and selectivity of the measurement method. Using aptamer-based diagnostic elements in combination with nanomaterials to modify the surface of the electrochemical sensors has received much attention [28,29,30]. Previous studies have shown that using aptamer sequences specific to each medicinal substance enables the unique diagnosis in addition to improving the sensitivity of the measurement method [31,32]. Up to now, the epirubicin analysis was done along with other structurally similar compounds but it was not possible to measure it with high selectivity [33,34]. In this work, we introduce a new electrochemical aptasensor based on a gold electrode modified with carbon nano-anion and aptamer as a new sensor for the electrochemical determination of epirubicin in biological samples. The gold electrode surface was modified with a thiolated aptamer, which can selectively bind to the epirubicin molecules. The main difference between this work and the others is attributed to bonding ox-CNOs and MB to the self-assembled aptamer to boost the electron transfer and consequently enhances the sensitivity and selectivity. The addition of ox-CNOs to the modifier layer of increased the negative charge on the electrode surface so attracted more the positively charged MB ions onto the aptasesnor. Therefore, the aptasesnor sensitivity was improved towards the epirubicin. The capability of the method for analysis of the epirubicin in blood serum is evaluated.

## 2. Experimental Methods

### 2.1. Materials and Apparatus

In this work, the thiolated aptamer was purchased from BIONEER, South Korea. The sequence of the purchased thiolated aptamer is SH-5′ ACCATCTGTGTTAAGGGGTAAGGGGTGGGGGTGGGTACGTCT3. Diamond nanoparticles purchased from Merck were consumed to prepare CNOs. Other materials used, which include sodium hydroxide, sulfuric acid, ethanol, nitric acid, sodium bicarbonate, Tris-HCL, methylene blue (MB), sodium chloride, and 6-hydroxy-1-hexanethiol (MCH), were received from Merck. The epirubicin, daunorubicin, methotrexate, and toremifene drugs were obtained from Tofigh Daru. Electrochemical measurements were performed with a μ-Autolab electrochemical device using Nova software (Version 2.1.4). FT-IR device model Perkin Elmer, Field-Emission scanning electron microscope (FE-SEM) model TESCAN MIRA 3, and X-ray spectrometer (XRD) were used to characterized the synthesized CNOs.

### 2.2. Synthesis of CNOs

CNOs with good conductivity were synthesized by a straightforward method using diamond nanoparticles. For this purpose, 1.0 g of diamond nanoparticles was placed in a furnace under argon gas for one hour at a temperature of 1150 °C. Then, the sample was placed in a furnace under an ambient atmosphere for one hour at a temperature of 450 °C to remove amorphous carbons to prepare CNOs. A mixture solution of sulfuric acid and nitric acid was poured into a flask containing 1.0 g of CNOs and refluxed at 80 °C for 5 h to functionalize CNOs (ox-CNOs). It was then centrifuged, and the pH was adjusted with sodium bicarbonate; after it reached to pH = 7, it was washed with water and ethanol and kept at 60 °C for 12 h.

### 2.3. Preparation of the Electrochemical Aptasensor

First, the Au electrode was polished with a slurry of aluminium with a particle size of 5 μm, and it was washed with water and placed in a three-electrode cell system containing 1 M sulfuric acid solution. Afterwards, ten consecutive scans of the cyclic voltammetry in the potential range of −0.4 to 1.2 V at a scan rate of 100 mV/s were applied to the Au electrode surface. Next, the Au electrode was placed in the thiolated aptamer solution with a concentration of 1 μM in the Tris-HCl buffer solution containing 20 M NaCl for 120 min to form a layer of aptamer at the Au electrode surface due to the hybridization of the gold and thiol. Subsequently, the electrode was washed with an aqueous solution of NaCl and MgCl_2_. The Au electrode covered by a thiolated aptamer was immersed in a 2 mM MCH solution for 60 min to obtain a single layer of aligned aptamer, Au/Apt. This step led to the attachment of MCH to the active sites of the Au electrode that did not occupied by aptamer. Therefore, the remained active sites on the Au electrode were blocked to prevent the nonspecific adsorption of the analyte. Then, the Au/Apt electrode was placed in a phosphate buffer solution (pH = 7.4) containing epirubicin for 40 min to adsorb the target molecules on the electrode surface. After that, the Au/Apt/epirubicin electrode was immersed in a phosphate buffer solution (pH = 7.4) containing ox-CNOs for 180 min to achieve the Au/Apt/epirubicin/ox-CNOs electrode. Finally, Au/Apt/epirubicin/ox-CNOs electrode was put in the 120 μM MB solution for 30 min. the final modified electrode was named as Au/Apt/epirubicin/ox-CNOs/MB. The aptasensor free of target molecule was also prepared in the same way without the step of epirubicin adsorption. Finally, the prepared aptasensors were washed with water. Electrochemical measurements were performed by the differential-pulsed voltammetry (DPV) method, and quantitative measurements were conducted based on the current difference of the modified electrode in presence and absence of the epirubicin according to Equation (1).

Δ*I* = *I* (Apt) − *I* (Apt-epirubicin) (1)

## 3. Result and Discussion

### 3.1. Characterization

**Figure 1:**
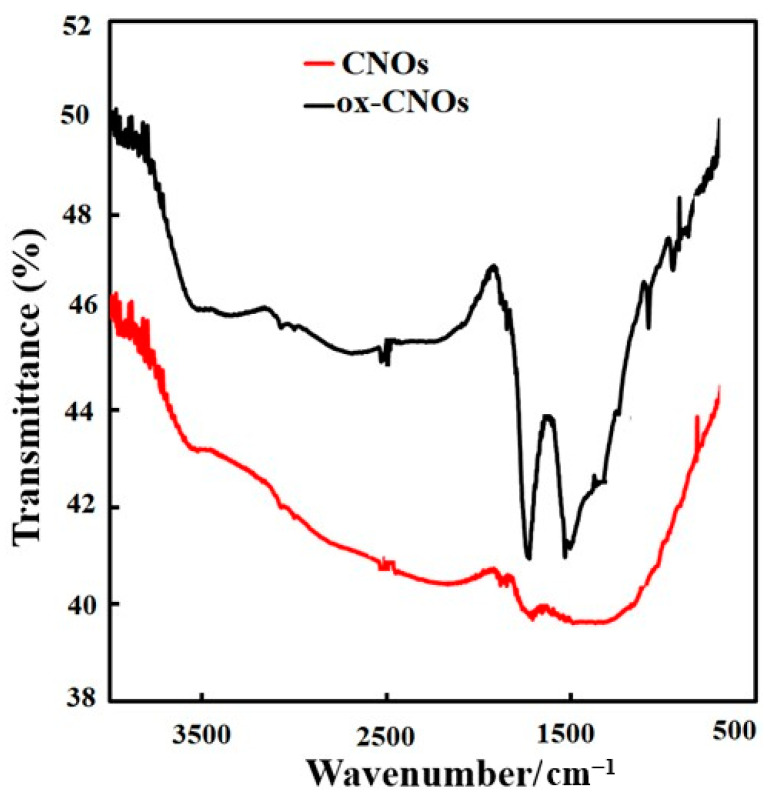
FT-IR spectra of CNOs and ox-CNOs.

The CNOs and ox-CNOs functional groups were investigated by an infrared spectrometer, Figure 1. The sharp peak observed at 1738 cm^−1^ is related to the stretching vibrations of the C=O group. The intensity of this peak became very high when CNOs were oxidized indicating that CNOs have been well oxidized. Also, the band observed in the region from 1510 to 1580 cm^−1^ is related to carboxyl and carbonyl groups. Some peaks with low intensity can also be seen in ox-CNOs, which are related to COOH groups that confirm the successful oxidation of CNOs.

**Figure 2:**
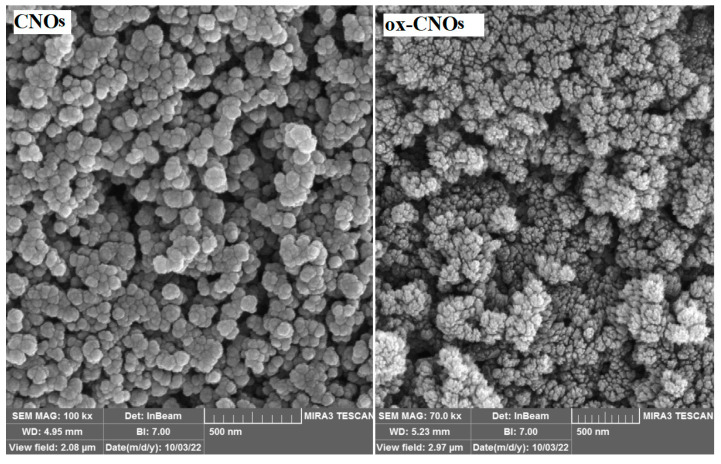
FE-SEM image of CNOs and ox-CNOs.

**Figure 3:**
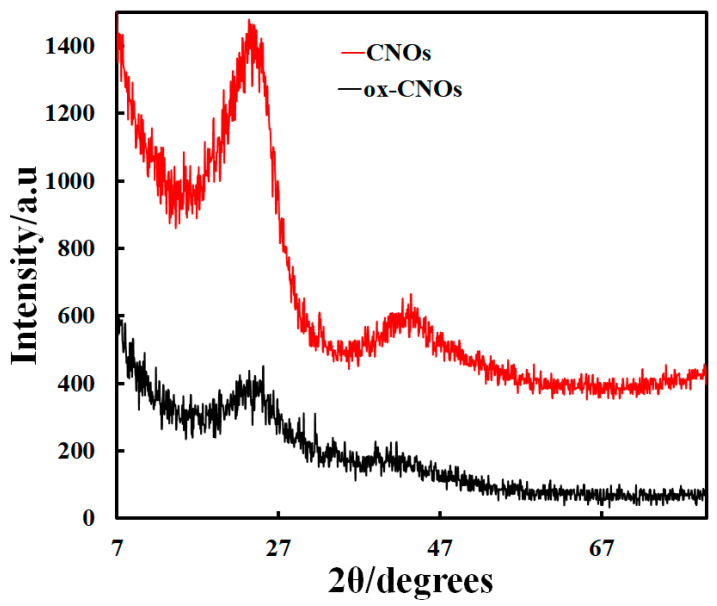
XRD pattern of CNOs and ox-CNOs.

FE-SEM images for CNOs and ox-CNOs nanoparticles were given in Figure 2. For CNOs, spherical nanoparticles with a relatively uniform particle size can be observed. The ox-CNOs show smaller particles out of the spherical state accumulated in a needle-shaped state. Figure 3 depicts the XRD patterns of CNOs and ox-CNOs. Broad peaks at 2θ = 24.3 and 44 are visible for CNOs. After oxidation of CNOs, the broadening of mentioned peaks occurred and a significant reduction in their intensity was observed.

### 3.2. Electrochemical Performance of Electrodes

**Figure 4:**
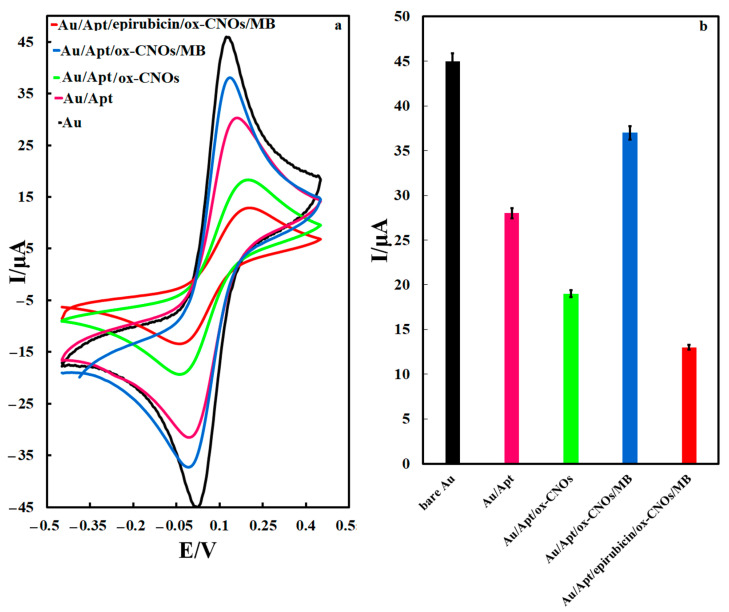
(**a**) CVs of different electrodes in 1 mM K_3_[Fe(CN)_6_]/K_4_[Fe(CN)_6_] as the probe solution, (**b**) oxidation current value of the probe solution on the surface of different electrodes.

**Figure 5:**
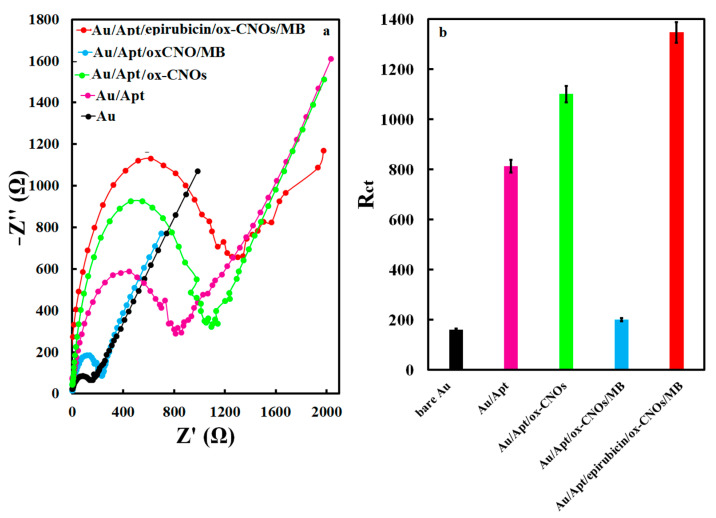
(**a**) Nyquist plots of different electrodes in 1 mM K_3_[Fe(CN)_6_]/K_4_[Fe(CN)_6_] solution and (**b**) the obtained R_ct_ value of different electrodes.

The cyclic voltammetry method in the potential range of −0.4 to 0.4 V was used to investigate the performance of the prepared electrodes towards the 1 mM solution of the K_3_[Fe(CN)_6_]/K_4_[Fe(CN)_6_] as probe, Figure 4. At the surface of the unmodified Au electrode, the electrochemical probe solution showed an oxidation/reduction redox couple with a potential difference of 0.11 V. By inserting the thiolated aptamer on the surface of the Au electrode, the oxidation/reduction current related to the probe solution decreased. The potential difference of the redox couple was more significant than the unmodified Au electrode. This phenomenon is due to the blocking of the Au electrode surface by the aptamer, which prevents the presence of probe ions on the electrode surface, so the oxidation/reduction reaction was not performed well. With the insertion of the ox-CNOs, the peak current related to the oxidation/reduction of the probe decreased while the potential difference increased. This performance is due to the negative charge of ox-CNOs, which prevented the negative ions of the probe from reaching the electrode surface and as a result reduced the electron transfer rate, decreased the current and increased the potential difference of redox peaks of the probe. Inserting the MB on the electrode surface increased the current and decreased the potential difference indicating negatively charged ox-CNOs absorbed positively charged MB ion well. After attachment of the epirubicin to the aptamer, the current reduction and the potential difference increment was observed again. This indicates the successful fixation of the epirubicin molecules on the aptamer, which reduced the electron transfer rate. The electrochemical impedance spectrometer in the frequency range of 10 mHz to 100 kHz in the ferri/ferrocyanide solution was also checked to investigate the electrode surface response after each step of the modification process. The Nyquist diagrams of the Au, Au/Apt, Au/Apt/ox-CNOs, Au/Apt/ox-CNOs/MB, and Au/Apt/epirubicin/ox-CNOs/MB electrodes in the probe solution were shown in Figure 5. The results showed the charge transfer resistances of 160, 813, 1101, 200, and 1347 Ω on the surface of Au, Au/Apt, Au/Ap/ox-CNOs, Au/Apt/ox-CNOs/MB, and Au/Apt/epirubicin/ox-CNOs/MB, respectively. By connecting the aptamer to the surface of the Au electrode, the charge transfer resistance enhanced, which is due to the non-conductivity and electrochemical inactivity of the aptamer strands. Also, the existence of ox-CNOs increased the charge transfer resistance because they limited the diffusion rate of probe ions towards the electrode surface. However, the charge transfer rate increased in the presence of MB. Moreover, the diameter of the semicircle part of Nyquist plots increased in the drug’s presence, indicating an increase in charge transfer resistance. These results confirmed the successful fabrication of the electrochemical aptasensor.

### 3.3. Optimization

**Figure 6:**
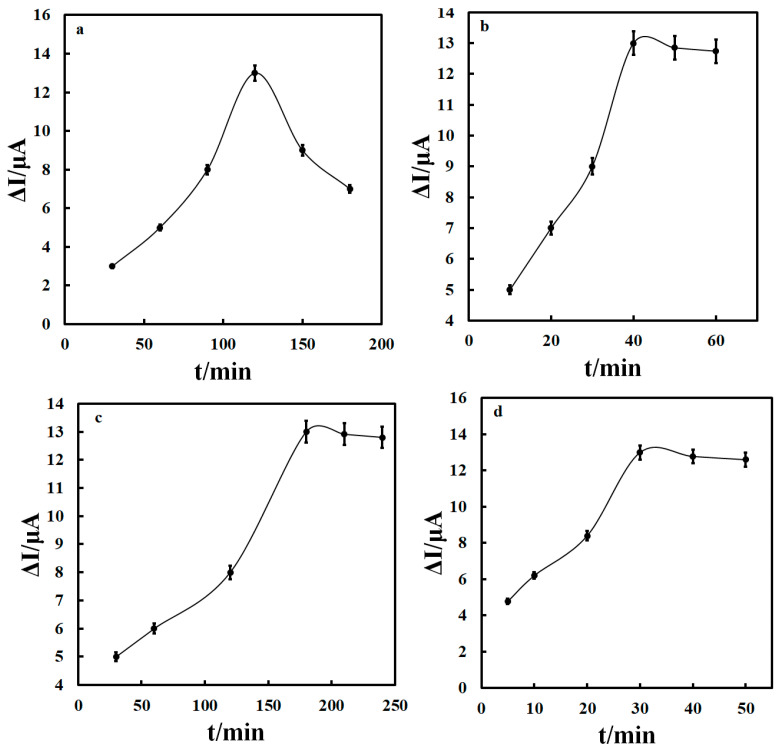
Optimization: (**a**) time required for attachment of aptamer to the Au electrode surface, (**b**) duration of the drug-aptamer interaction, (**c**,**d**) the period of the interaction between the aptamer and ox-CNO and MB, respectively.

Effective parameters, including duration of time required for placement of the aptamer on the Au electrode surface, duration of drug-aptamer interaction, the period of the interaction between the aptamer and ox-CNOs, and MB were investigated to access the best response from the prepared aptasensor. The current changes in the presence of aptamer and drug for each of the parameters are given in Figure 6. Studying the incubation time of aptamer on the Au electrode surface is the most important parameter that plays a vital role in the successful fabrication of the electrochemical aptasensor, so here different periods for the absorption of a self-assembled layer of aptamer on the Au electrode surface were investigated, Figure 6a. Noticeably, the difference between the oxidation peak current of MB at Au/Apt/epirubicin/ox-CNOs/MB and Au/Apt/ox-CNOs/MB enhanced by increasing the time of the Au electrode incubation in the aptamer strands from 30 to 120 min. By further increasing the incubation time up to 180 min, no sensible change was observed in the aptasensor response towards epirobicin. This result revealed that the 120 min incubation time is adequate to completely cover all accessible sites on the Au electrode surface with aptamer strands. The incubation time of the Au/Apt into the 15 nM epirubicin was checked between 10 and 60 min. The results showed that the final aptamer sensing increased up to 40 min and remained constant after that. Forty min was selected as the optimal time for epirobicin absorption on the aptasensor, Figure 6b. In addition, the insertion of ox-CNOs on the Au/Apt/epirubicin surface increased the aptasensor response. The ox-CNOs insertion time was evaluated in the range of 30–240 min. The aptasensor response increased by time duration up to 180 min and remained almost constant for further time, Figure 6c. The electrode immersion into the MB solution led to the insertion of MB along the ox-CNOs between aptamer strands and as a result boosting the sensing sensitivity. The best time for the MB insertion was 30 min, Figure 6d.

### 3.4. Analytical Activity of Epirubicin

**Figure 7:**
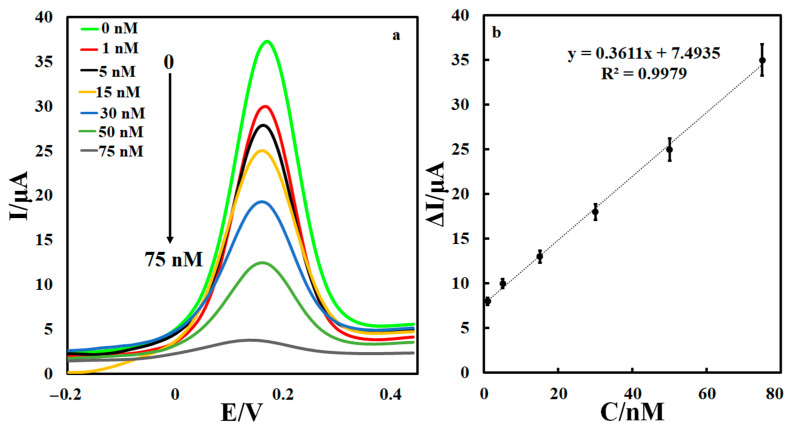
(**a**) Electrochemical aptasensor responses towards different concentrations of epirubicin, (**b**) calibration curve.

The epirubicin sensing by the developed electrochemical aptasensor in a phosphate buffer solution with pH = 7.4 was investigated in the concentration range of 1–75 nM under optimal conditions. Differential pulse voltammograms (DPVs) of aptasensor based on optimized procedure were recorded for different concentrations of epirubicin. A decrement of peak current for MB oxidation was observed with increment of the drug concentration. A linear relationship was resulted between the epirubicin concentration and aptasensor response in the absence and the presence of the epirubicin in the range of 1–75 nM, Figure 7. An excellent linear relationship was obtained with a line slope of 0.36 μI/nM. According to the equation of LOD = 3 Sb/m, a detection limit equal to 0.33 nM was calculated.

A comparison of the performance of the electrochemical methods reported for epirubicin analysis with the aptasensor prepared in this work was given in Table 1. The results showed that the detection limit obtained in this work is comparable to previous researches indicating suitability of the prepared electrochemical aptasensor for epirubicin measurement.

**Figure 8:**
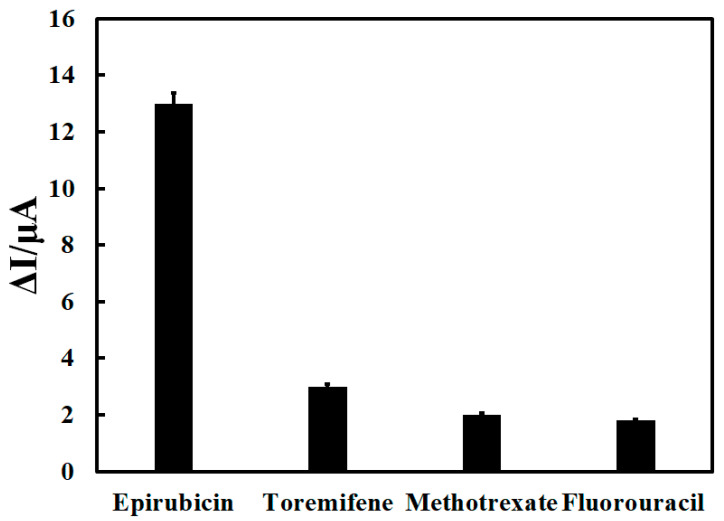
Investigating the selectivity of the prepared electrochemical aptasensor in 15 nM epirubicin measurement.

The prepared electrochemical aptasensor was also used to measure the pharmaceutical compounds of daunorubicin, methotrexate, and toremifene to check the selectivity. The current changes obtained for these drugs showed that the prepared electrochemical aptasensor is unsuitable for measuring other drug compounds with anticancer capabilities, so it has excellent selectivity for measuring epirubicin, Figure 8. The reproducibility of the prepared aptasensor was determined by preparing four electrochemical aptasensors with the same method to measure epirubicin. The results of measuring epirubicin with those four aptasensors have a relative standard deviation of 2.34%, which indicates the good reproducibility of the aptasensor fabrication. Also, four consecutive DPV measurements were performed by every Au/Apt/epirubicin/ox-CNO/MB in a buffer solution. The results showed a relative standard deviation of 3.42 for this experiment, which indicates the appropriate repeatability of this electrochemical aptasensor.

The epirubicin value in a plasma sample was measured with the prepared electrochemical aptasensor using the standard addition method. The results are given in Table 2. The recovery percentage obtained for measuring epirubicin in the concentration range of 5, 10, and 15 nM was 96–98%, which indicated the very good capability of the prepared aptasensor for measuring epirubicin in real samples.

## 4. Conclusions

Here, a very sensitive electrochemical aptasensor based on a Au electrode covered by a single layer of self-assembled aptamer having suitable reproducibility and the ability to measure epirubicin in real samples was prepared. The MCH strands were attached to the unoccupied sites of the Au electrode by aptamer to prevent the nonspecific adsorption of the epirubicin. Methylene blue and ox-CNOs were used to track and increase the sensitivity of the prepared aptasensor. The synthesized CNOs were chemically oxidized to produce carbonyl and carboxylic functional groups, which improve and facilitate their adsorption to the aptamer strands. The increment of negatively charged functional groups on the CNOs boosted the adsorption of the positively charged MB leading to more sensitive aptasensor response. Functionalized CNOs nanoparticles paired with MB improved the performance of the aptasensor and increased the sensitivity of the aptasensor. Our funding showed that with raising epirubicin concentration, the electrochemical response of the fabricated aptasensor under optimal empirical conditions linearly enhanced in the concentration range of 1–75 nM, and the detection limit of 0.33 nM was resulted for epirubicin. One of the most important features of this prepared aptasensor is its low cost and easy preparation, which can quickly measure trace amounts of epirubicin.

## Figures and Tables

**Table 1:** The performance comparison of developed sensors for the epirubicin measurement.

Modified Electrode	Technique	Linear Range (nM)	LOD (nM)	Ref.
NiFe_2_O_4_/AuNPs/SPCE	DPV	700–3600	5.3	[13]
Ce-ZnO/GCE	DPV	10–600	2.3	[33]
DNA/GCE	DPV	50–500	10	[35]
AuNPs/Fe_3_O_4_/SiO_2_/SPCE	DPV	70–2100	40	[36]
DNA/AuNPs/SPCE	DPV	40–2000	10	[37]
Au/Apt/epirubicin/ox-CNO/MB	DPV	1–75	0.33	This Work

**Table 2:** Epirubicin determination in plasma sample.

Sample	Added (nM)	Found (nM)	Recovery (%) (n = 3)	RSD %
Plasma	0	-	-	-
	5	4.85	97.00	4.19
	10	9.76	97.60	4.31
	15	14.80	98.66	3.95

## Data Availability

Not applicable.
